# Unique Epitopes Recognized by Antibodies Induced in Chikungunya Virus-Infected Non-Human Primates: Implications for the Study of Immunopathology and Vaccine Development

**DOI:** 10.1371/journal.pone.0095647

**Published:** 2014-04-22

**Authors:** Yiu-Wing Kam, Wendy W. L. Lee, Diane Simarmata, Roger Le Grand, Hugues Tolou, Andres Merits, Pierre Roques, Lisa F. P. Ng

**Affiliations:** 1 Singapore Immunology Network, Agency for Science, Technology and Research (A*STAR), Biopolis, Singapore, Singapore; 2 NUS Graduate School for Integrative Sciences and Engineering, National University of Singapore, Singapore, Singapore; 3 CEA, Division of ImmunoVirology (SIV), Institute of Emerging Diseases and Innovative Therapies (IMETI), Fontenay-aux-Roses, France; 4 Université Paris-Sud 11, UMR E1, Orsay, France; 5 Groupe d’Etude en Préventologie (GEP), Villenave d’Ornon, France; 6 Institute of Technology, University of Tartu, Tartu, Estonia; 7 Department of Biochemistry, Yong Loo Lin School of Medicine, National University of Singapore, Singapore, Singapore; University of Massachusetts Medical Center, United States of America

## Abstract

Chikungunya virus (CHIKV) is an *Alphavirus* that causes chronic and incapacitating arthralgia in humans. Although patient cohort studies have shown the production of CHIKV specific antibodies, the fine specificity of the antibody response against CHIKV is not completely defined. The macaque model of CHIKV infection was established due to limitations of clinical specimens. More importantly, its close relation to humans will allow the study of chronic infection and further identify important CHIKV targets. In this study, serum samples from CHIKV-infected macaques collected at different time-points post infection were used to characterize the antibody production pattern and kinetics. Results revealed that anti-CHIKV antibodies were neutralizing and the E2 glycoprotein, Capsid, nsP1, nsP3 and nsP4 proteins were targets of the anti-CHIKV antibody response in macaques. Furthermore, linear B-cell epitopes recognized by these anti-CHIKV antibodies were identified, and mapped to their structural localization. This characterizes the specificity of anti-CHIKV antibody response in macaques and further demonstrates the importance of the different regions in CHIKV-encoded proteins in the adaptive immune response. Information from this study provides critical knowledge that will aid in the understanding of CHIKV infection and immunity, vaccine design, and pre-clinical efficacy studies.

## Introduction

Chikungunya virus (CHIKV) was first described during an epidemic in 1952 in Tanzania, East Africa as the causative agent of Chikungunya fever (CHIKF) [1], [2]. CHIKV belongs to the genus *Alphavirus* of the family *Togaviridae* and is an enveloped virus with a single-stranded positive-sense RNA genome [3]. The 12kb RNA genome is capped at the 5′ end and polyadenylated at the 3′ end and consists of two open reading frames coding for four non-structural proteins (nsP1–4), three major structural proteins (Capsid, E1, and E2) and two small cleavage products (E3 and 6K) [3], [4]. The E1 and E2 glycoproteins form heterodimers that associate as trimeric spikes on the virion surface while E3 and 6K were demonstrated to act as helper proteins in the budding and maturation process of the virion envelope [5]–[7].

In the last decade, multiple CHIKF epidemics have occurred in East Africa, the Indian Ocean Islands, and many parts of South East Asia [8]–[13]. More recently, new episodes of CHIKF have been reported in the Americas, further broadening the geographical spread of the disease [14]. The *Aedes* species of mosquito has been the major arthropod vector associated with CHIKV transmission to humans [15]. CHIKV infection usually leads to the development of CHIKF and is characterized by an abrupt onset of fever, headache, fatigue, nausea, vomiting, rash, myalgia, and severe arthralgia. Similar to other arthralgia-causing arbovirus infections, a fraction of patients developed chronic symptoms lasting from several weeks to months [1], [2], [15].

Currently, there are no licensed vaccines or antiviral drugs against CHIKV infection for human use. Therapy for CHIKV infection is often limited to supportive care [4]. Despite the development of several animal models, few have met the requirement to be used in pre-clinical study to assess potential therapeutics.

Recent epidemiological data showed the increasing importance of antibody-mediated protection against CHIKV [16]–[19], highlighting the feasibility of using anti-CHIKV antibodies as therapeutics or as a prophylactic treatment [20]. However, information about the exact target of the adaptive immune response either in human or in animal models remains limited, although B-cell epitopes have been identified within the E1/E2 glycoproteins [17], [21].

Due to the close lineage relationship between humans and macaques, macaque models of CHIKV infection have been developed [22]–[24]. These models allow comparison of the adaptive immunity between humans and macaques. Furthermore, information obtained from macaque studies will be valuable for the design of future therapeutics.

In this study, we aimed to investigate the kinetics and specificity of anti-CHIKV antibodies induced after experimental infection in cynomolgus macaques (*Macaca fascicularis*). In addition to the anti-E2 glycoprotein responses, we also identified new linear B-cell epitopes that were recognized by anti-CHIKV antibodies from CHIKV-infected macaques. These epitopes can potentially be used for future development of vaccine candidates.

## Materials and Methods

### Ethics Statement

All animals were handled in strict accordance with good animal practice as defined by the European directive 63/2010/EU and in accordance with recommendations of the Weatherall report. Four to six-year-old cynomolgus macaques (*Macaca fascicularis*) were imported from the international accredited breeding facilities from Mauritius (negative for SIV, STLV, herpes B virus, filoviruses, SRV-1, SRV-2, measles, dengue virus and CHIKV) and were housed in a BSL3 facility (Permit Number A 92-032-02), in accordance with Office for Laboratory Animal Welfare (OLAW, USA; #A5826-01) standards at the CEA in accordance with the French national regulation under the number B-92-032-02 for animal use, under the number 2005-69 for macaque breeding. Animals are fed daily and monitored closely by caretakers reporting directly to the veterinarians in charge of the animal facilities. All studies were reviewed and approved by the regional animal care and use committee in accordance with European directive 63/2010/EU: “Comité regional d’éthique pour l’expérimentation animale Ile-de-France Sud”, Fontenay aux Roses, decision #07_012.

Cell lines originally purchased from American Tissue Culture Collection (ATCC), such as human embryonic kidney (HEK 293T, ATCC CRL-3216) and baby hamster kidney (BHK21, ATCC CCL-10) cells, were adhered to recommended ethics approvals and standards.

### Cells and Virus Isolates

HEK 293T cells were cultured in Dulbecco’s Modified Eagle Medium (DMEM) supplemented with 10% Fetal Bovine Serum (FBS) (Gibco, Invitrogen). CHIKV isolates (LR2006 OPY-1, IMT and SGP11) used in this study were originally isolated from two French patients returning from Reunion Island during the 2006 CHIKF outbreak [25], [26], and from a Singaporean admitted to the National University Hospital in 2008 [27] respectively. CHIKV LR2006 OPY-1 clone was rescued from corresponding infectious cDNA clone as previously described [28]. Virus stocks (IMT and SGP11) used for *in vitro* studies were prepared via numerous passages in Vero-E6 cultures, titered, washed and pre-cleared by centrifugation before storage at −80°C [29]. LR2006-OPY1 was isolated from patient serum in Marseille and passaged three times in Vero-E6 culture. Virus stocks were produced following a single passage in BHK21 cells for experimental infection of macaques.

### Macaque CHIKV Infection Model

The animals were first sedated with ketamine chlorhydrate (10 mg/kg; Rhone-Mérieux) and were inoculated with 10^5^ up to 10^8^ pfu of CHIKV via the saphenous vein [22]. Clinical examinations were carried out as described previously [22], and temperature and weight of the macaques were recorded 15 minutes after sedation. Sera were collected by bleeding from the femoral vein before the first CHIKV inoculation and on a daily basis after the infection until 16 days post-infection (dpi). CHIKV-infected macaques were kept for a maximum of up to 180 dpi.

### *In vitro* Neutralization

Neutralizing activity of antibodies from CHIKV-infected macaque samples were tested in triplicates and analyzed by immunofluorescence-based cell infection assay in HEK 293T cells. Amount of CHIKV virions corresponding to MOI 10 were mixed with heat-inactivated macaque serum (1∶100–1∶800 dilutions), and incubated for 2 hours at 37°C with gentle agitation in a thermo-mixer. Virus-antibody mixtures were then added to HEK 293T cells seeded in 96-well plates and incubated for 1.5 hours at 37°C. Medium was removed, and cells were replenished with DMEM medium supplied with 5% FBS and incubated for 6 hours at 37°C before fixation with 4% paraformaldehyde followed by immunofluorescence quantification using the Cellomics ArrayScan V. Percentage of infectivity was calculated according to the equation: % Infectivity = 100× (% responder from sero-neutralization group/% responder from virus infection group).

### CHIKV Immunoblot

Proteins from CHIKV virions were detected using modified techniques previously described [29], [30]. For reducing samples preparation, sample containing IMT and SGP11 virions were boiled for 5 minutes at 100°C in Laemmli buffer supplemented with 2% SDS and 1 mM DTT. Non-reducing samples were prepared in Laemmli buffer supplemented with 2% SDS but without DTT and were not boiled. SDS migration buffer was used for electrophoresis.

Proteins from CHIKV virions were separated by 10% SDS-PAGE respectively, and transferred to nitrocellulose membrane at 180 mA for 45 min in transfer buffer (24 mM Tris, 77 mM glycine, 20% methanol) using semi-dry transfer method. The membranes were blocked overnight in blocking buffer (TBST supplemented with 5% dry milk and 3% FBS), followed by 1 hour incubation at room temperature with either antigen-specific rabbit serum (1∶2000), or macaque serum samples diluted (1∶2,000) in blocking buffer. Antigen-specific sera against nsP1, nsP2, nsP3, nsP4, Capsid, E2 and E1 proteins were raised in rabbits by passive immunization. The appropriate HRP-conjugated anti-rabbit IgG or anti-monkey IgG secondary antibodies were then added and incubated for 1 hour, followed by chemiluminescence detection using ECL Plus detection reagents (Amersham Biosciences). Blots were exposed to films (Pierce, Thermo Scientific) and developed.

### Virion-based ELISA

Polystyrene 96-well microtiter plates (MaxiSorp, Nunc) were coated with purified CHIKV (20,000 infectious virions per µl in PBS; 50 µl per well). Wells were blocked with PBS containing 0.05% Tween-20 and 5% non-fat milk (PBST-milk), and plates were incubated for 1.5 hours at 37°C. Serum samples were then diluted 1∶2,000 in PBST-milk and incubated 1 hour at 37°C. HRP-conjugated rabbit anti-monkey IgG (Alpha Diagnostic) antibodies were used to detect macaque antibodies bound to virus-coated wells respectively. Reactions were developed using TMB substrate (Sigma-Aldrich) and terminated by Stop reagent (Sigma-Aldrich). Absorbance was measured at 450 nm. Non-infected macaque serum was used as negative controls. ELISA readings were done in duplicates and the values were plotted as mean ± standard error mean (SEM).

### Peptide-based ELISA

Biotinylated peptide library consisting of 18-mer overlapping peptides (Mimotopes) were generated from sequence alignments of different CHIKV amino acid sequences as previously described [17], [29]. Peptides were dissolved in dimethyl sulphoxide (DMSO) to obtain a stock concentration of approximately 15 µg/mL. All the peptides were screened in triplicates. Briefly, streptavidin-coated plates (Pierce) were first blocked with 1% sodium caseinate (Sigma-Aldrich) diluted in 0.1% PBST (0.1% Tween-20 in PBS), before coating with peptides diluted at 1∶1,000 in 0.1% PBST and incubated at room temperature for 1 hour on a rotating platform. Plates were then rinsed with 0.1% PBST before incubation with CHIKV-infected macaque serum samples (1∶2,000) diluted with 0.1% PBST for 1 hour. Plates were rinsed and then followed by incubation with the respective anti-monkey IgG antibodies conjugated to HRP diluted in 0.1% blocking buffer for 1 hour at room temperature to detect for peptide bound antibodies. Reaction was detected with TMB substrate solution (Sigma-Aldrich) and terminated with sulphuric acid (Sigma-Aldrich). Absorbance was read at 450 nm in a microplate autoreader (Tecan). Peptides are considered positive if absorbance values are higher than the mean ±3 standard deviation (SD) values of non-infected macaque serum controls. Data are presented as mean ± SEM.

### Computational Modeling

Structural data of the E2 glycoproteins were retrieved from PDB (id: 3N44) and visualized using the UCSF CHIMERA software [31]. Solvent excluded molecular surfaces were generated with the help of MSMS package [32]. Structures of Capsid, nsP1, nsP3 and nsP4 sequences were predicted separately using individual I-TASSER queries, and visualized using UCSF CHIMERA software [33], [34].

### Statistics

All data are presented as mean ± SEM or SD. Differences in responses among groups at various time points and between groups and controls were analyzed using appropriate tests (Mann-Whitney *U* tests). A two-sided *P* value of less than 0.05 was considered to be statistically significant.

## Results

### Antigen Recognition by CHIKV-infected Macaques

**Figure 1:**
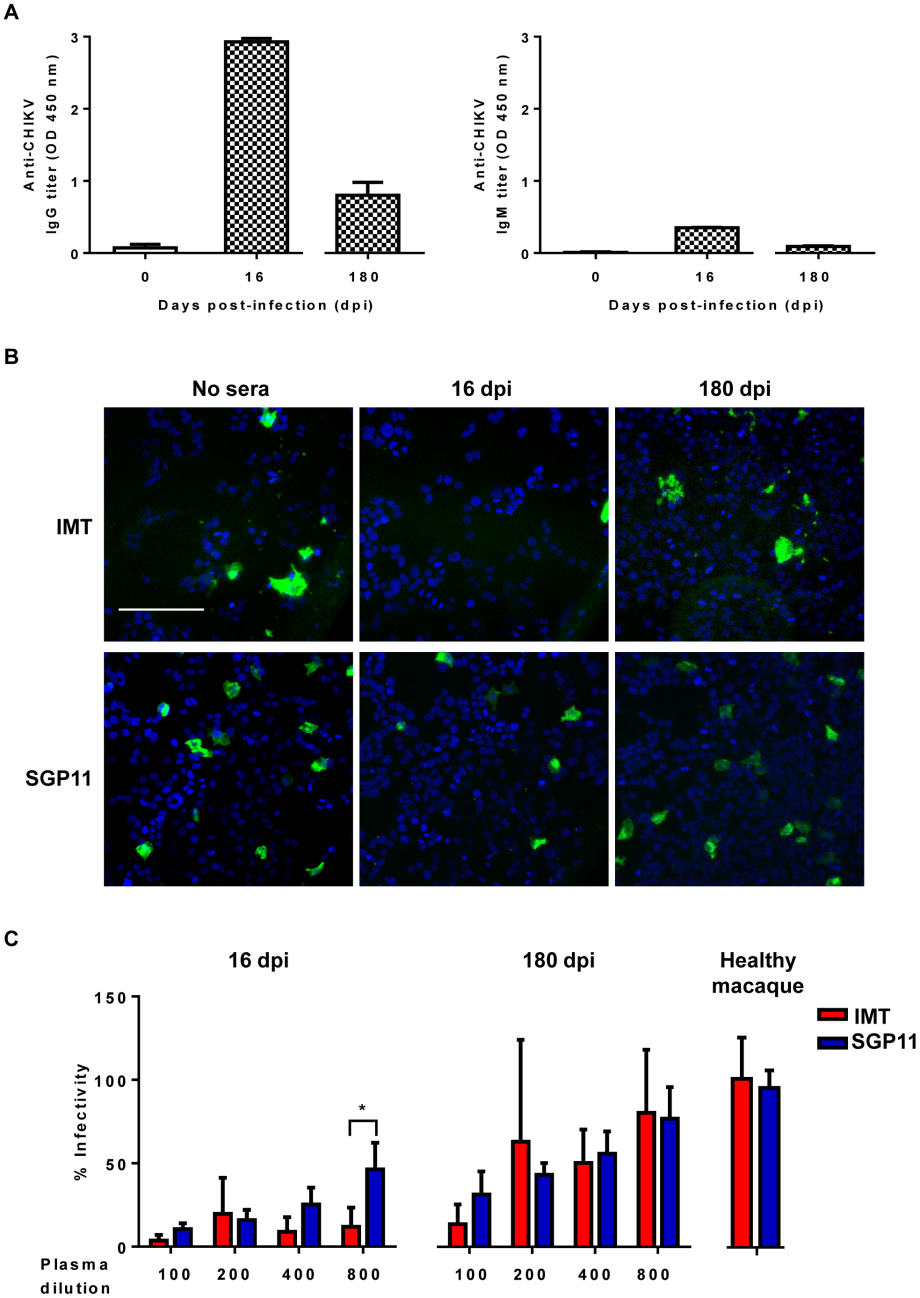
Antibody profiles of sera from CHIKV-infected macaques. *A,* Virus-specific IgG and IgM antibody titers in serum samples, at a dilution of 1∶2,000 were determined by ELISA using purified CHIK virions. Serum samples from CHIKV-infected macaques (n = 1–3) were collected at 16 and 180 dpi and subjected to virion-based ELISA, using 96-well plates pre-coated with purified CHIKV (IMT) virions. Sera from non-infected macaques were used as negative controls. Data are presented as mean ± SD and representative of 2 independent experiments with similar results. *B,* Visualization of CHIKV by immunofluorescence in CHIKV-infected cultures after seroneutralization. Virus samples were pre-incubated with heat-inactivated sera from CHIKV-infected macaques collected at 16 and 180 dpi, before being added to HEK 293T cells. Infection without pre-incubation with sera (No sera) was used as a control. Analysis was performed at 6 hours post-infection (hpi). Scale bar in white: 50 µm. Representative microscopic images for each treatment condition (macaque sera dilution at 1∶800) are shown. *C, In vitro* neutralizing activity of sera from CHIKV-infected macaques. Samples (Healthy macaque sera, 16 and 180 dpi) were tested against IMT or SGP11 viruses, in triplicates at a dilution 1∶800 for healthy macaque sera; between 1∶100 and 1∶800 for CHIKV-infected macaque sera. Results are representative of 3 independent experiments, presented as mean ± SD, and expressed as percentage of control infection. *p<0.05, Mann-Whitney *U* test.

In order to characterize the CHIKV antibody response, we obtained serum samples taken at two post-infection time-points from CHIKV (LR2006 OPY-1)-infected macaques. The total IgG present in the serum was quantified by a virion-based ELISA assay using CHIKV isolate from La Reunion (IMT) [25], [29]. High levels of CHIKV-specific IgG antibody responses were detected at 16 dpi (Figure 1A) confirming previous observations [17]. Even though the IgG levels decreased over time, they were still detectable at 180 dpi (Figure 1A). To determine if the anti-CHIKV antibodies were neutralizing, *in vitro* infection of HEK 293T cells with different CHIKV isolates (IMT and SGP11) was carried out in the presence of sera from CHIKV-infected macaques (Figure 1B and 1C) [29]. Serum samples from CHIKV-infected macaques contained antibodies that displayed neutralizing activity when assessed in sero-neutralization assays [16], [17] with the highest neutralizing efficiency for samples taken at 16 dpi (Figure 1C). Interestingly, a stronger neutralizing activity against IMT was observed when compared to SGP11 for samples at 16 dpi, at a dilution of 1∶800 (Figure 1C). This is not unexpected as these macaques were experimentally infected with the LR2006 OPY-1 isolate that is more closely related to the IMT isolate. The presence of neutralizing antibodies in the sera of CHIKV-infected macaques was sustained till 180 dpi, although less efficiently than sera at 16 dpi (Figure 1C).

**Figure 2:**
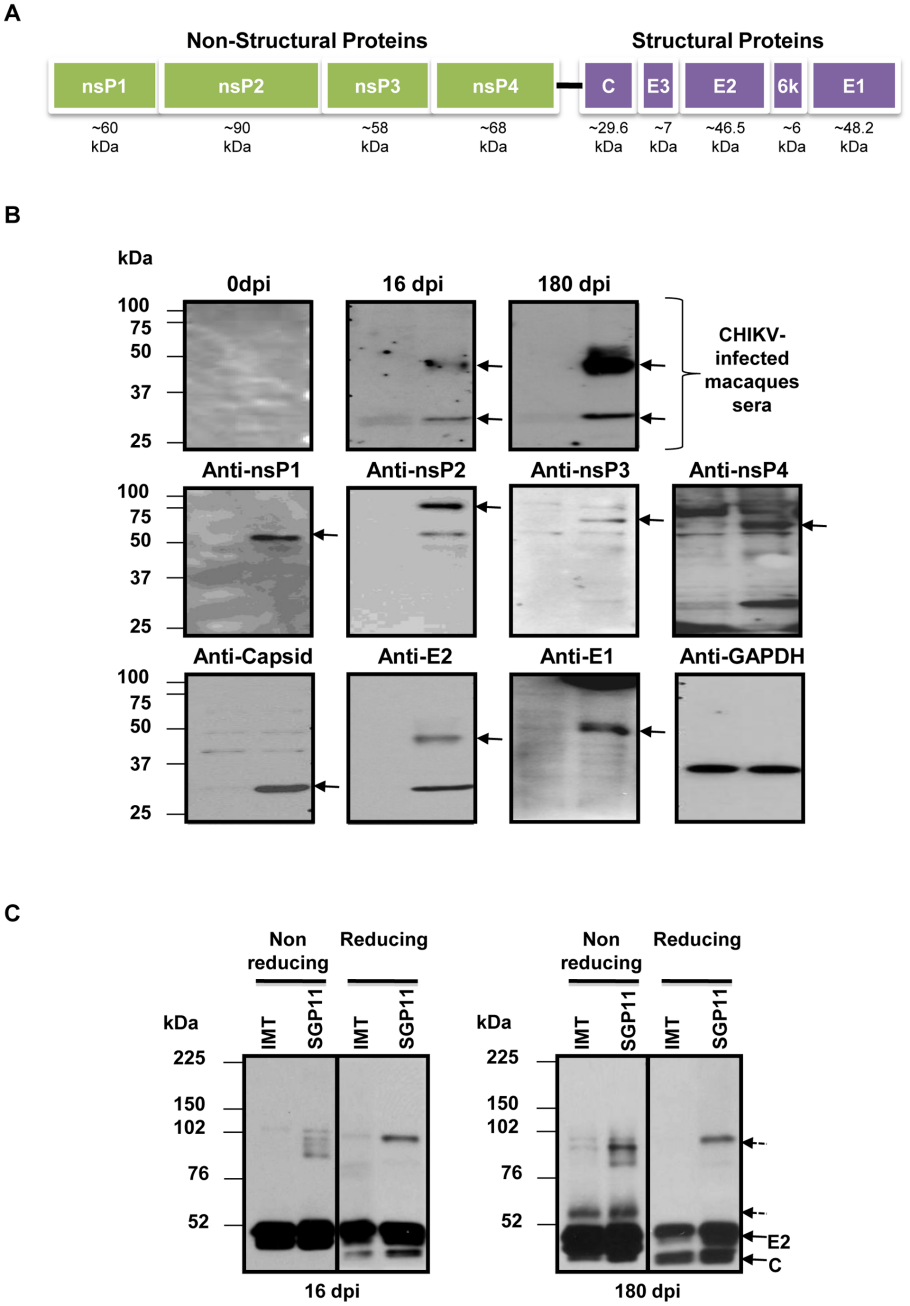
Anti-CHIKV antibodies demonstrate polymorphic epitope recognition against different CHIKV isolates. *A,* Schematic representation of the coding regions of CHIKV genome. *B,* Total cell lysates were prepared from CHIKV-infected 293T cells and mock-infected 293T cells. Lysates were subjected to SDS-PAGE gel electrophoresis (Left lane – mock-infected lysates, right lane – CHIKV-infected lysates) and probed with sera from CHIKV-infected macaques at a dilution of 1∶2,000, followed by HRP-conjugated anti-monkey IgG secondary antibodies. The arrows at the top panel indicate the CHIKV antigens (Capsid and E2) detected by antibodies in the sera of CHIKV-infected macaques. Arrows at the middle panel (non-structural proteins) and the lower panel (structural proteins) indicate CHIKV proteins detected by corresponding CHIKV antigen-specific rabbit sera performed at a dilution of 1∶2,000 followed by HRP-conjugated anti-rabbit IgG secondary antibodies. Housekeeping protein GAPDH was probed with a specific anti-GAPDH antibody (Biolegend) as an indicator for loading control. Sizes of molecular weight markers are indicated in the left part of the diagram. *C,* Purified CHIKV virions (IMT and SGP11) were prepared under reducing (100°C, 5 min+DTT) or non-reducing conditions and subjected to SDS-PAGE gel electrophoresis, then probed with sera from CHIKV-infected macaques at a dilution of 1∶2,000, followed by secondary HRP-conjugated anti-monkey IgG. Sizes of molecular weight markers are indicated on the left part of the diagram. Non-annotated arrows represent the presence of disulfide bonds dependent conformational epitopes recognized by antibodies from the sera of CHIKV-infected macaques.

To better define the antigenic recognition profile of the antibodies from CHIKV-infected macaques, we used CHIKV-infected cell lysates to determine which CHIKV antigens were recognized by the macaque antibodies (Figure 2A). None of the non-structural proteins (nsPs) were recognized by antibodies in the serum samples taken at 16 or 180 dpi (Figure 2B). The serum contained antibodies against E2 glycoprotein and Capsid protein (Figure 2B and S1). Anti-E2 and anti-Capsid antibodies persisted till 180 dpi. In addition, CHIKV virions were purified from supernatant of cells infected with CHIKV isolates from Reunion Island (IMT) [25], or from Singapore (SGP11) [27]. Protein extracts were prepared under reducing or non-reducing conditions as described [29], [30], and were then assessed by immunoblot assays using serum samples from CHIKV-infected macaques (Figure 2C). This first screen revealed that these serum samples contained antibodies recognizing multiple proteins from two isolates of CHIKV. A stronger reactivity against proteins prepared under non-reducing conditions compared to proteins prepared under reducing conditions indicated that the serum samples contained antibodies recognizing both linear and disulfide bonds dependent conformational epitopes (Figure 2C) [35]. Similar reactivity was observed between two closely related CHIKV isolates (IMT and SGP11) that have more than 99% amino acid sequence similarity [36], suggesting that the two isolates present epitopes that are common or similar in part and are recognized by a fraction of the antibodies from macaques infected with the third closely related isolate of CHIKV.

### Mapping of CHIKV Protein Regions Recognized by the Macaque Antibodies

**Figure 3:**
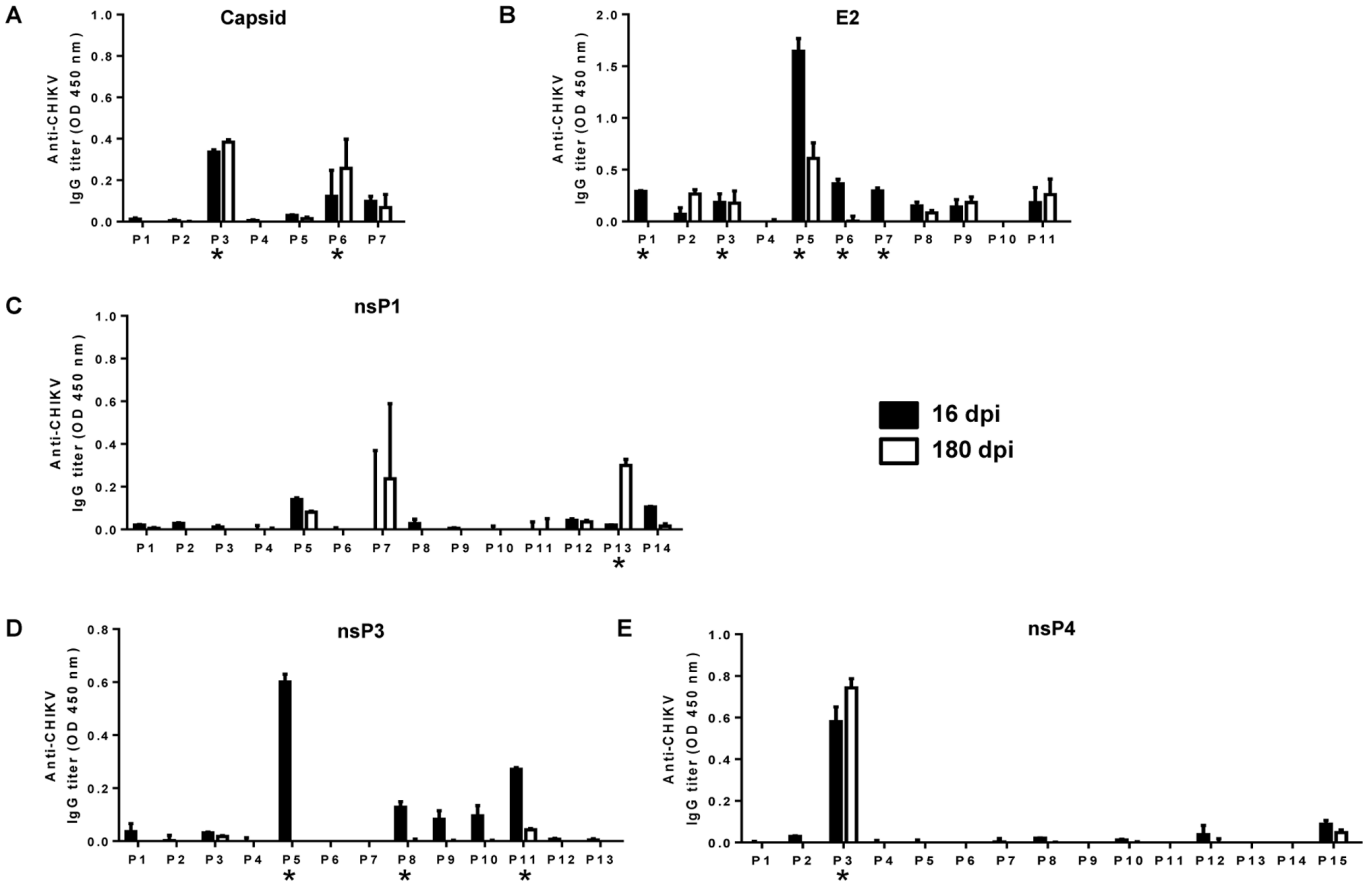
Mapping of CHIKV B-cell epitopes within CHIKV proteome. Sera from CHIKV-infected macaques (16 and 180 dpi) were diluted 1∶2,000 and subjected to peptide-based ELISA with a peptide library covering the CHIKV proteome, using pooled peptides (named P1, P2, etc) from the structural (*A,* Capsid, *B,* E2) and non-structural (*C,* nsP1, *D,* nsP3 and *E,* nsP4) proteins. Sera from non-infected macaques were used as negative controls. *Pooled peptides were considered to contain positive linear B-cell epitopes when OD values obtained with sera from CHIKV-infected macaques were above mean +3 SD of the OD values obtained with sera from non-infected macaques. Data are presented as OD values obtained using sera from infected macaques, minus the OD values obtained using sera from non-infected macaques, for the corresponding pooled peptides. Data represent an average of two independent experiments (mean ± SD).

ELISA assays were performed using a biotinylated peptide library (Mimotopes) with minimized non-specific binding. The library consisted of 18-mer overlapping peptides covering the whole CHIKV proteome. Pooled peptides were screened with pooled serum samples taken at two different time-points [17], [29]. Several peptide pools were identified that contained linear CHIKV B-cell epitopes recognized by the serum antibodies (Figure 3, peptide pools that positively bound serum antibodies are marked with *). Results showed that there were two such peptide pools for the Capsid protein (Figure 3A), five for the E2 glycoprotein (Figure 3B), one for the nsP1 protein (Figure 3C), three for the nsP3 protein (Figure 3D) and one for the nsP4 protein (Figure 3E). The recognition pattern of antibodies from the two different time-points was not identical. No peptide pools containing linear B-cell epitopes were detected for the remaining proteins (nsP2, E3, 6K and E1) (data not shown).

**Figure 4:**
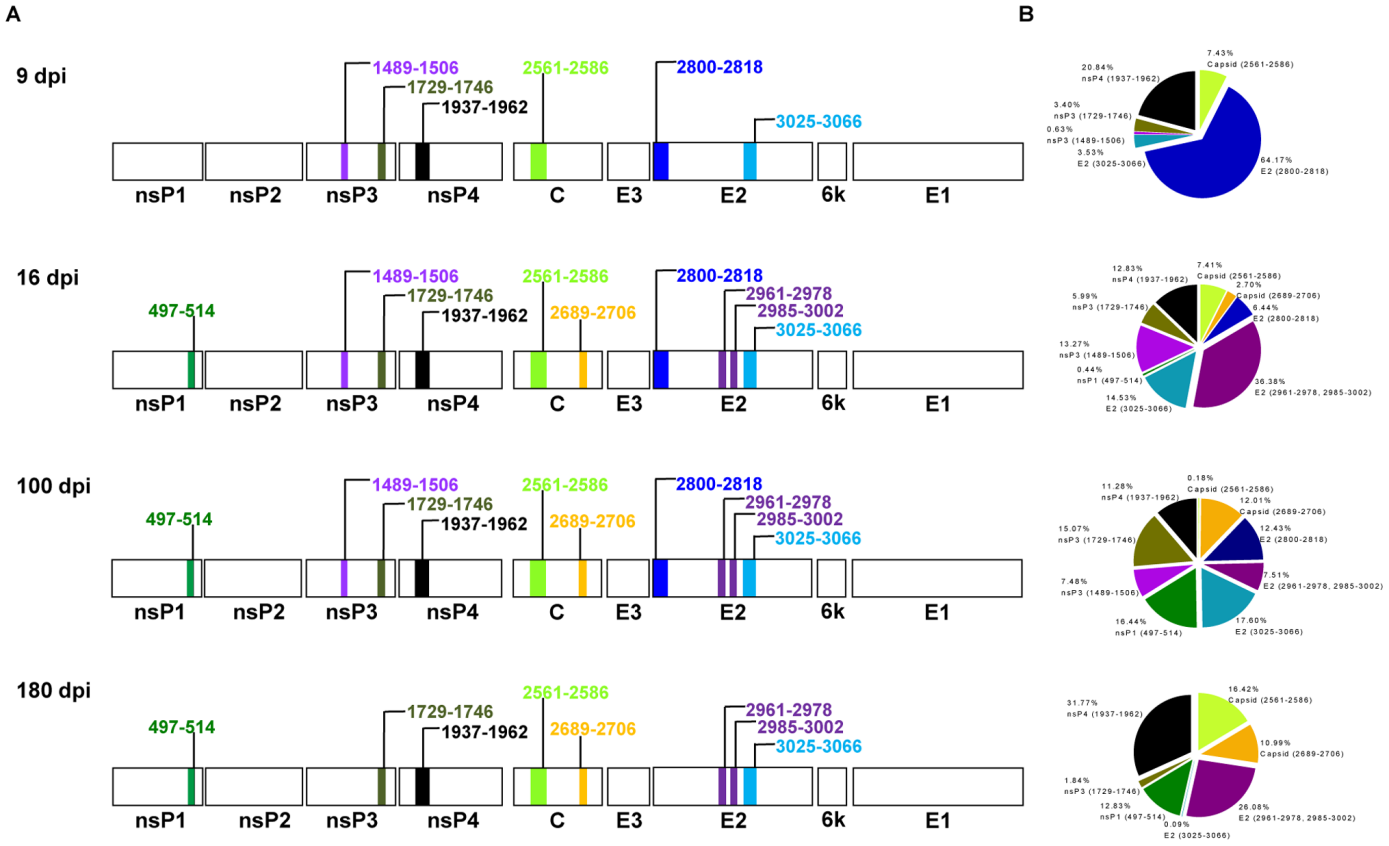
Analysis of anti-CHIKV antibodies recognizing linear B-cell epitopes. *A,* Serum samples from CHIKV-infected macaques (n = 1–3) were collected at 9, 16, 100 and 180 dpi. Peptide-based ELISA covering the CHIKV proteome, encompassing the non-structural and structural proteins, was performed with sera diluted at 1∶2,000. Regions of amino acid sequence corresponding to the identified linear B-cell epitopes are indicated on the genome organization schematic diagram. *B,* The percentage of antibody recognition for various CHIKV epitopes is indicated on the pie-charts. The percentage was calculated according to the equation: % antibody recognition = 100 x (OD values from pooled peptide group/sum of OD values from all pooled peptide groups).

To study the kinetics of the establishment of the adaptive immunity in CHIKV-infected macaques, serum samples from macaques infected intravenously with the Reunion Island strain (LR2006 OPY-1) were collected over a 6-month period [22]. Anti-CHIKV antibody profiles were followed longitudinally through different post-infection phases (Figure 4). All the macaques presented with fever, rash and lymphopenia, and exhibited signs of inflammation as indicated by cytokine patterns and biochemistry assays, from 1 to 6–8 dpi [22]. The 9 dpi time point in the acute phase was the earliest time point when specific anti-CHIKV antibodies were detected (Figure 4). At 9 dpi, 90% of the total signal came from IgM that was no longer detectable at 16 dpi, in the early convalescent phase. Furthermore, at 16 dpi, the first detectable levels of IgG were also observed. Importantly, the later time-points of 100 and 180 dpi in the recovery phase allowed the plateau and persistence of the IgG specific response to be assessed, while levels of IgM were no longer detectable (data not shown). Next, to define the location of the linear B-cell epitopes, the complete set of single peptides from each of the antibody-binding peptide pools identified in Figure 3, was screened with serum samples. The positions of the epitope sequences within the non-structural and structural proteins are illustrated in the schematic diagrams (Figure 4A). The two recognized regions for the Capsid protein are located at the N- and C-terminus of the protein while the epitopes for the E2 glycoprotein are distributed along the entire protein. On the other hand, antibodies from the macaque serum samples failed to recognize any regions in the 6K protein and E1 glycoprotein. Comparison of the reactivity between the two time-points showed that antibodies recognized the linear epitopes in Capsid, and in the immunodominant E2 region in a concerted manner (Figure 4B).

Taken together, results from the peptide-based ELISA assay were consistent with the immunoblot assays, confirming the recognized regions in the E2 glycoprotein, and the Capsid protein.

### Structural Localization of the Antigenic Regions Recognized by Macaque Antibodies

**Figure 5:**
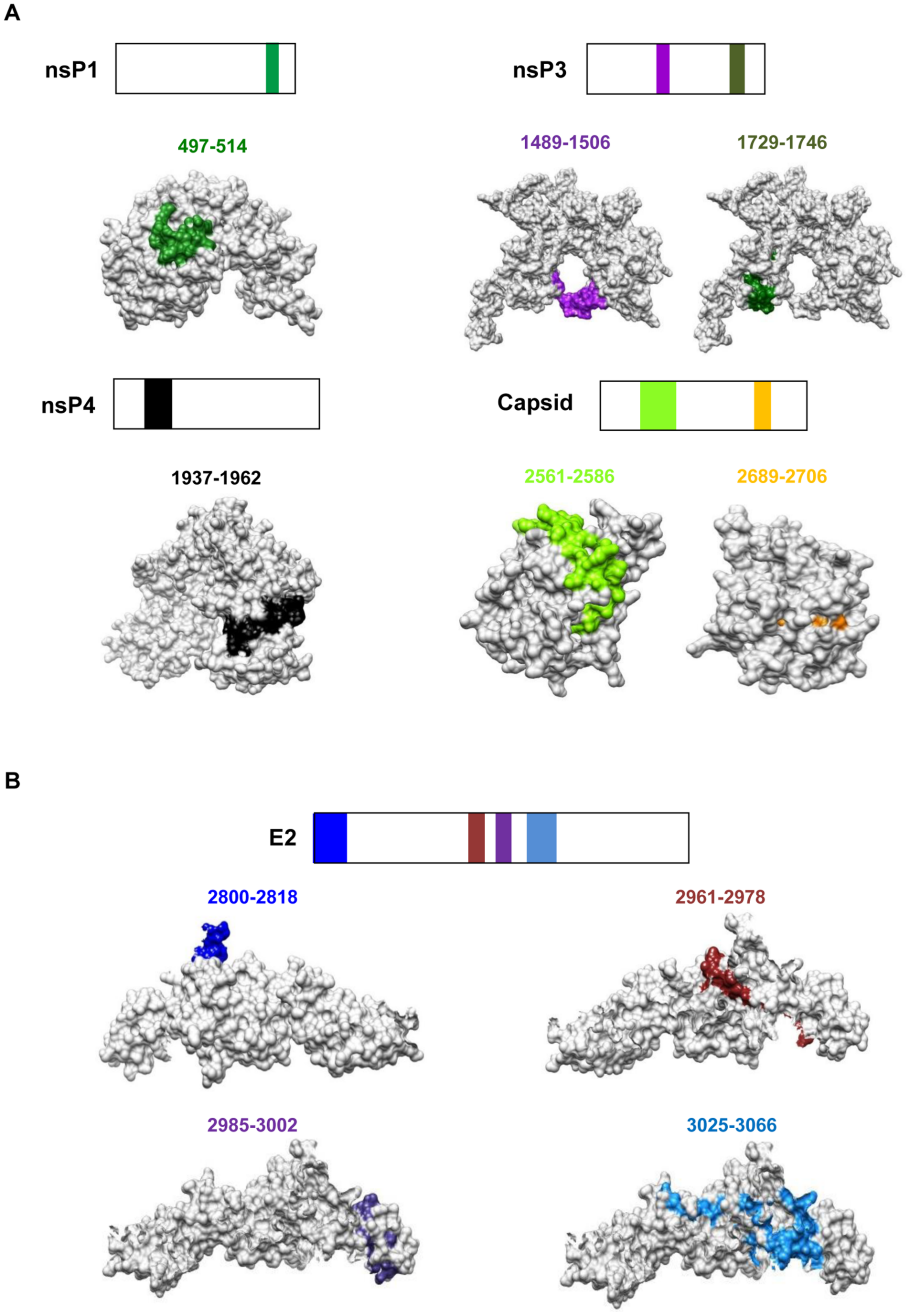
Localization of linear CHIKV B-cell epitopes within the CHIKV proteome. *A,* Schematic representation of identified B-cell epitopes in nsP1, nsP3, nsP4 and Capsid protein. *B,* Schematic representation of identified B-cell epitopes in E2 glycoprotein. Epitopes in the E2 glycoprotein were located based on structural data retrieved from PDB number: 3N44. Epitopes in the Capsid, nsP1, nsP3 and nsP4 proteins were located based on structures predicted by I-TASSER server.

Epitope-containing sequences were mapped onto the available three-dimensional (3D) crystal structure of the E2 glycoprotein (PDB number 3N44), or predicted 3D structures of the Capsid, nsP1, nsP3 and nsP4 proteins as described previously (Figure 5) [29]. One of the epitope-containing regions of the Capsid protein (amino acids 2561–2586) was located on the surface of the protein, while the other was concealed in the folded protein (Figure 5A). Furthermore, the two recognized regions of the nsP3 protein were located on the surface of the protein (Figure 5A).

Similar analyzes for the E2 glycoprotein revealed that one out of four recognized regions mapped onto the surface of the protein (Figure 5B). Majority of the epitopes clustered in the middle of the protein and were embedded in the E2 glycoprotein.

## Discussion

Defining an ideal animal model for CHIKV studies remains a constant challenge. In order to understand the immunology and pathophysiology of CHIKV infections, an animal model with disease presentations closely similar to humans is preferred.

In order to study the temporal pattern of CHIKV B-cell epitopes in detail, experimental infections were performed in macaques with serum samples collected over 6 months. Linear determinants were targeted since they are more easily identifiable in a medium-throughput approach. Although conformational epitopes could also contribute to the anti-CHIKV antibody response, these determinants are not explored due to the limitation of detection by the linear peptides screen described here. Results showed two structural proteins (Capsid protein and E2 glycoprotein) and three nonstructural proteins (nsP1, nsP3 and nsP4 proteins) contained linear epitopes that were recognized by macaque anti-CHIKV antibodies. Experimental infection of macaques induced antibodies not only against the structural proteins, but also against several non-structural proteins. The important role of antibodies against structural proteins has been previously demonstrated in sero-neutralization and protection assays [17], [18], [29]. Whether antibodies targeted against the non-structural proteins also offer protection during CHIKV infection will require further investigation.

Studies on antibody responses against various CHIKV proteins using plasma from patients obtained at different post-infection time-points have shown that the N-terminal region of E2 glycoprotein provides long-lasting anti-CHIKV antibody response during the whole course of disease [17], [29]. Specifically, high levels of antibodies from CHIKV-infected patients were targeted against a linear B-cell epitope called E2EP3 during the acute phase of CHIKV infection [17]. Here, E2EP3 is one of the major B-cell linear epitopes recognized by antibodies from the sera of all CHIKV-infected macaques during the early phase of disease (amino acids 2800–2818, Table 1). This verifies that the presence of anti-E2EP3 antibodies is a common marker for early CHIKV infection in both humans and macaques [17]. Previous findings that the E2 and E3 glycoproteins, Capsid and nsP3 proteins were specifically detected during the convalescent and recovery phase of human disease [29] suggest that the pattern of B-cell epitope recognition by anti-CHIKV antibodies alters as the disease progresses.

**Table 1:** Comparison of human and macaque CHIKV B-cell epitopes.

CHIKV protein	Identified B-cell epitope (human)^a^	Amino acid^b^	Identified B-cell epitope (macaque)	Amino acid^b^
nsP1			AEEEREAELTREALPPLQ	497–514
nsP2				
nsP3	GVNSVAIPLLSTGVYSGG	1433–1450	IQMRTQVELLDEHISIDC	1489–1506
	FGASSETFPITFGDFNEGEIESLSSELLTFGDFLPGEVDDLTDSDWSTCSCSDTDDELLDRAGGYIFS	1801–1867	TVPVAPPRRRRGRNLTVT	1729–1746
nsP4			AAIIQRLKRGCRLYLMSETPKVPTYR	1937–1962
Capsid	KDIVTKITPEGAEEW	2721–2735	PPKKKPAQKKKKPGRRERMCMKIEND	2561–2586
			RPIFDNKGRVVAIVLGGA	2689–2706
E3	LLQASLTCSPHRQRR	2785–2799		
E2	STKDNFNVYKATRPYLAHC	2800–2818	STKDNFNVYKATRPYLAHC	2800–2818
	TDGTLKIQVSLQIGIKTDDSHDWTKLRYMDNHMPADAERAGL	2841–2882	ATTEEIEVHMPPDTPDRT	2961–2978
	LTTTDKVINNCKVDQCHAAVTNHKKW	3009–3034	GNVKITVNGQTVRYKCNC	2985–3002
	HAAVTNHKKWQYNSPLVPRN AELGDRK * GKIHIPFPLAN	3025–3058	HAAVTNHKKWQYNSPLVPRN AELGDRK * GKIHIPFPLANVTCR	3025–3066
	PTVTYGKNQVIMLLYPDHPTLLSYRN	3073–3098		
	PTEGLEVTWGNNEPYKYWPQLSTNGT	3121–3146		
	LLSMVGMAAGMCMCARRRCITPYELTPGATVPFL	3177–3210		
6K				
E1				

a Regions of B cell epitopes found that are common to both human and macaque are underlined.

b The numbers correspond to the amino acid positions along the CHIKV viral genome. The first amino acid from nsP1 is annotated as 1.

*K – Amino acid lysine at position 252 within the CHIKV E2 glycoprotein.

The acid-sensitive region (ASR) of E2 glycoprotein has been suggested to play a role in regulating CHIKV particle generation and virulence *in vivo* [37], [38]. Moreover, the functional role of the ASR in regulating E1/E2 glycoprotein conformational changes suggests that the ASR could be a target for neutralizing antibodies [39]. Our previous results showed that natural CHIKV infection induced antibodies against only one ASR epitope (amino acids 3025–3058, Table 1). These antibodies were detected during the recovery phase (2 to 3 months post illness onset) [29]. However, anti-CHIKV antibodies against two ASR epitopes (amino acids 2961–2978, and 3025–3066, Table 1) were detected in the sera of experimentally infected macaques. Furthermore, these two ASR epitopes were immunodominant from the early convalescent to recovery phase, contrary to the immunodominance of the E2EP3 epitope observed in natural human CHIKV infection [17], [29]. The observation that one of the ASR epitopes (amino acids 3025–3058) is important in both natural human infection and experimental macaque infection, suggests that this epitope could be useful for vaccine development.

In addition, our results showed that antibody recognition of the E2 glycoprotein changes throughout the course of disease in the experimentally infected macaques. This could be due to the spatial positions of the B-cell epitopes on the native form of the E1/E2 glycoprotein complex. Differential induction of neutralizing antibodies against exposed or hidden B-cell epitopes could contribute to antibody-mediated clearance during the entire course of disease [40]. This is important in CHIKV vaccinology since it underscores the importance of eliciting a broad antibody response targeting exposed and hidden B-cell epitopes that would be sufficient to cover most of the important antigens for virus neutralization.

It has been established that patient antibodies from a Singapore cohort have stronger binding capacity to the CHIKV SGP11 isolate than the IMT isolate due to different epitope sequences between the two isolates, which influences epitope-antibody binding capacity [29]. Here, we further demonstrated this phenomenon in the non-human primate model. Infection of macaques with the LR2006-OPY1 isolate, which encodes K_252_ in the E2 glycoprotein, strongly induced anti-CHIKV antibodies against a particular linear B-cell epitope (amino acids 3025–3066) at 16 dpi. In line with this, we observed significantly stronger neutralizing activity against the IMT isolate (encoding K_252_ in E2) compared to the SGP11 isolate (encoding Q_252_ in E2) (Figure 1C). These data complement a separate study that showed stronger neutralizing antibody response in animals infected with the ECSA strain (DHS-4263) compared to animals infected with the West African strain (37997) [24]. Paradoxically, the difference in neutralizing capacity observed here was lost in macaque sera taken at 180 dpi. Incidentally, sera taken at this time point also had relatively low levels of anti-CHIKV antibodies against the same B-cell epitope (amino acids 3025–3066). These observations highlight the importance of incorporating various components in antigen preparation for CHIKV vaccine development. Epitope sequences covering all the relevant isolates from various geographical regions will be favored in order to offer complete coverage against CHIKV infections globally.

Only four common linear B-cell epitopes in the E2 glycoprotein could be identified by antibodies obtained from patients and CHIKV-infected mice [18], while two epitopes were common between patients and CHIKV-infected macaques (Table 1, underlined sequences). Recent studies have also demonstrated that CHIKV-infected patients reacted sero-positively to the E2 glycoprotein [17], [29], [41]. These observations were further supported by other studies with B-cell epitopes identified along the E2 glycoprotein by mouse and human monoclonal antibodies [21], [42]–[44]. Contrastingly, linear epitopes along the E1 glycoprotein were detected only by antibodies from CHIKV-infected mice. Comparatively, antibodies obtained from experimentally-infected mouse models in other alphaviruses have also identified epitopes from both the E1 and E2 glycoproteins [18], [40], [45]–[48]. Divergence in epitope recognition in the various animal models suggests the existence of species-related differences in the humoral response upon virus infection.

Only a few vaccine candidates against CHIKV have been tested in macaques [49], [50]. All other studies were assessed in mouse models [51]–[54]. In these models, the protective effect was closely associated to the generation of neutralizing antibodies, but only few have reported on the exact epitopes involved [50], [52]. Our previous work in CHIKV-infected macaques showed that natural infection led to viral persistence in all macaques tested up to 90 dpi [22] despite a robust innate immune response that was protective *in vitro*. As such, a comprehensive study on the exact targets of infection- versus vaccination-induced antibodies will provide more information about antibody-mediated protection against the chronic CHIKV infection observed in macaques.

## Supporting Information

Figure S1 (PDF)
