# Supplementary material for: Unique Epitopes Recognized by Antibodies Induced in Chikungunya Virus-Infected Non-Human Primates: Implications for the Study of Immunopathology and Vaccine Development
Source: PLoS One. 2014 Apr 22;9(4):e95647. doi: 10.1371/journal.pone.0095647 (PMC3995782; doi:10.1371/journal.pone.0095647)
Supplement: Figure S1 — (PDF) [file pone.0095647.s001.pdf]

Structural Proteins

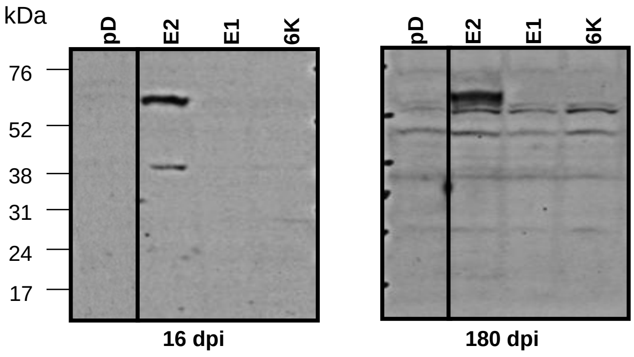

Figure S1. Antigen recognition of CHIKV-infected macaques' sera. Codon-optimized cDNA clones encoding the CHIKV proteome were synthesized (Genescript Corporation) and sub-cloned into pDisplay (pD) expression vector (Invitrogen). Recombinant CHIKV proteins were expressed in HEK 293T cells and purified. Total cell lysates were prepared from transiently expressed E2 glycoprotein (E2), E1 glycoprotein (E1) and 6K protein. Lysates were subjected to SDS-PAGE gel electrophoresis and probed with CHIKV-infected macaques' sera at a dilution of 1:2,000, followed by secondary HRP-conjugated anti-monkey IgG. Control cell lysates were prepared from cells transiently transfected with pD plasmids.

Figure_S1 Kam_Lee *et al*., 2014
